# The Quality of Medical Care in the Conditions of the COVID-19 Pandemic, with Particular Emphasis on the Access to Primary Healthcare and the Effectiveness of Treatment in Poland

**DOI:** 10.3390/jcm10163502

**Published:** 2021-08-09

**Authors:** Magdalena Kludacz-Alessandri, Renata Walczak, Liliana Hawrysz, Piotr Korneta

**Affiliations:** 1 College of Economics and Social Sciences, Warsaw University of Technology, 09-400 Plock, Poland; 2 Faculty of Civil Engineering, Mechanics and Petrochemistry, Warsaw University of Technology, 09-400 Plock, Poland; 3 Faculty of Management, Warsaw University of Technology, 02-524 Warsaw, Poland; piotr.korneta@pw.edu.pl

**Keywords:** primary healthcare, COVID-19, access to healthcare, treatment effectiveness

## Abstract

Health has a significant influence on the quality of life of a society. The COVID-19 pandemic has forced many countries to implement restrictive measures to prevent its wider spread, including, inter alia, the introduction of remote healthcare in the form of teleconsultations. Therefore, there is the question of how such a change affects the quality of treatment and the primary healthcare of patients during the COVID-19 pandemic. The article aims to examine patient satisfaction with the access to primary healthcare and the effectiveness of treatment in a condition of remote medical care caused by the COVID-19 pandemic. We also analyse the impact of access to primary healthcare on the treatment effectiveness. Patient satisfaction was measured using a questionnaire assessing the quality of primary medical care. Of the 36 items studied, seven were related to the accessibility dimension and four were related to the treatment effectiveness dimension. Our results suggest that the treatment effectiveness and the access to primary healthcare services during the COVID-19 pandemic through telemedicine are quite highly rated by patients. Hence, further implementation of telemedicine in primary healthcare should improve the quality of lives of the wide society. We have also identified the access to primary healthcare has a considerable impact on the treatment effectiveness. Therefore, we recommend increasing the contact between patients and GPs via telemedicine under lockdown conditions.

## 1. Introduction

Quality of life (QOL) can be defined as an individual’s perception of his or her life status in terms of the cultural systems and values of life, and concerning personal goals, expectations, standards, and concerns [1]. Quality of life became established as a significant concept and target for research and practice in the fields of health and medicine. Understanding QOL is important for improving symptom relief, care and rehabilitation of patients. QOL is also used for identifying the range of problems that can affect patients. This kind of information can be communicated to future patients to help them anticipate and understand the consequences of their illness and its treatment. QOL is also important for medical decision-making because it is a predictor of treatment success and is therefore useful in diagnostics [2]. The studies carried out so far show that medical care significantly contributes to the improvement of patients’ QOL [3,4].

QOL indicators, aimed at measuring progress in a society, should reflect its multidimensionality and cover aspects contributing to life satisfaction. One of the indicated aspects is health. Health has already been defined by the World Health Organization (WHO) as “a state of complete physical, mental and social well-being, and not merely the absence of disease or infirmity” [5,6]. Poor health not only has the potential to shorten people’s life expectancy, but it can also worsen their quality of life. At the collective level, it hampers economic and social development, reducing the so-called “human capital” available to a society and generating additional costs. Thus, long and healthy life is an indicator of social prosperity and success and a QOL factor. This means that the improvement of the quality of life is very often seen as a desirable result of the provision of primary healthcare (PHC) [7].

Traditionally, the quality of life in the field of health sciences is also used as an outcome variable to evaluate treatment effectiveness [8]. Health-related indicators are also used in various studies to measure the quality of life, e.g., health and access to healthcare [9], deaths from cardiovascular diseases and government spending on healthcare [7]. On the other hand, some have suggested that the impact of health and medical care on the overall quality of life is rather small [10].

Health and well-being often depend on the quality of healthcare, which is defined as the degree to which health services for individuals and the population increase the likelihood of achieving desired health outcomes and are consistent with current professional knowledge [1]. The quality of healthcare is also defined as “the degree to which health services meet the needs, expectations and standards of medical care for patients, their families and other care recipients” [11]. These aspects are very often examined in patient satisfaction surveys [12,13,14]. Several terms are functioning interchangeably in the literature regarding healthcare quality, including health status, quality of life, quality of care and health-related quality of life (HRQoL) [5,15]. Most of the QOL research in medicine and healthcare is related to health, and HRQOL is becoming increasingly important in healthcare and clinical research [16].

The WHO suggests that quality of life covers several key areas known as “domains”. In the domain defined as “environment”, there is an area related to Health and social care, including the accessibility and quality dimension related to, inter alia, with effectiveness [17]. Overall, the goal of quality in healthcare is the continual improvement of the patient’s condition. To decipher whether best practice on healthcare quality has been achieved, the concepts of access and effectiveness are systematically discussed in every healthcare environment [18,19]. For instance the studies that analyzed the impact of quality of care on patients’ QoL have measured health and well-being in terms of access to health care, effective treatment and social care [20].

Health-related quality of life maximisation and to the pursuit to provide high-quality medical services are among the most important goals in the work of a family doctor and challenges for primary care organisations, especially in the context of limited treatment options during the COVID pandemic, where telemedicine is often the only possible form of patient care. The need to improve the quality of medical services also results from the legal and ethical obligations of the GP [1].

The scope of this study was deliberately narrowed to the primary healthcare. This was done due to the primary healthcare’s fundamental role in many healthcare systems around the world [21] and in the Polish healthcare system [22]. It was already postulated that countries with developed primary healthcare enjoy fewer hospital admissions, better outcomes of patient treatments and, consequently, lower overall healthcare expenditure [23]. As a result, many scholars claim that the main objective of any governmental health policy should be the improvement of the primary healthcare quality [24]. The role of the primary healthcare is especially critical for the Polish healthcare system, mainly because of the following: ageing Polish society, substantial shortages in medical personnel (doctors and nurses) and lower expenditure compared to other EU countries on average.

Studies on the quality of primary healthcare during the COVID-19 pandemic in Poland are limited. On the other hand, researchers were dealing with this issue in the field of selected specialist services. This particularly included studies on access to medical care during pregnancy [25], bariatric care [26], cancer patients care [27], medical and non-medical services for the elderly [28]. Researchers agree that the COVID-19 pandemic situation leads to a growing problem of limited or complete lack of access to treatment for specific groups of patients who are in need of special care [29,30,31]. The perception of medical services during the COVID-19 pandemic was also studied in the context of cancer care [27]. However, no studies on patient satisfaction on the access to primary healthcare and treatment effectiveness in Poland could be found.

The core value of primary healthcare is that a well-organised and effective system of PHC is able to respond to the vast majority (even as much as 80%) of health needs with relatively small funds. Appreciation of the role of primary healthcare was the aftermath of analyses determining the impact of individual factors on healthy societies [32]. Barbara Starfield [33] has proven beyond any doubt that the quality of the entire healthcare system depends much more on the level of primary healthcare development than on the overall expenditure for the healthcare system.

Quality in primary healthcare is defined as the combination of access to healthcare, treatment effectiveness, while the improvement of the population’s health and the access to medical care are considered as two important objectives related to the core activities of health systems [34,35]. QOL assessments related to primary healthcare access and effectiveness can benefit patients, clinicians, researchers, administrators, health organizations, and policymakers. As the effects of the COVID-19 pandemic are likely to persist, research into the accessibility and effectiveness of primary healthcare is becoming extremely important, particularly in the context of telemedicine and QOL [36]. To check if best practice on primary healthcare quality has been achieved, the concepts of access and effectiveness should be systematically discussed in every primary healthcare entity [18, 19].

Access is critical to the functioning of primary healthcare systems around the world. However, access to primary healthcare remains a complex concept as exemplified by the concept’s interpretations diversity by authors. In primary healthcare, access is often defined as access of a service, provider or institution, and is thus defined as the possibility or ease with which patients can use adequate medical services relative to their needs [37]. Some researchers tend to equate access to a delivery system (for example, distribution and volume regarding the medical workforce and medical facilities, availability of providers and health facilities). Others argue that access can best be assessed using performance indicators for a patient’s passage through the system, such as utilisation rates or satisfaction scores [24,38,39].

In terms of remote medical appointments, access (accessibility) is considered as the patient’s ability to receive primary healthcare [40]. Accessibility is also defined as a way of organising primary care resources to accommodate a wide range of patient opportunities to contact physicians and access to primary healthcare services. This includes the doctors’ working hours, consultation times, telephone services and a flexible system enabling having an appointment for medical consultation [41].

Effectiveness was recognised as an important dimension of primary healthcare quality, but the literature emphasises the difficulty of characterising the definition of effectiveness for the primary healthcare sector. For example, in a 2004 study using the Delphi method to establish operational definitions for different dimensions of primary healthcare quality, despite repeated efforts, it was impossible to find a concise operational definition of effectiveness to which all experts could agree [41].

In real life, the concept of effectiveness is used interchangeably with the terms efficacy and efficiency, which is not correct from a scientific point of view. These three concepts have been originally distinguished by Drucker in management sciences [42] in which they bear the following meanings:

- efficacy—the ability to produce the desired amount of the desired effect, i.e., success in achieving a specific goal;
- effectiveness—the degree to which the planned results, objectives or tasks are achieved as a result of an action, intervention or initiative aimed at achieving the desired effect, in ordinary, uncontrolled circumstances;
- efficiency—doing things in the most economical way. It is the ratio of performance to the inputs of any system [43].

In the healthcare sector, efficacy is defined as the possibility of a beneficial change (or the therapeutic effect) as result of an intervention (e.g., drug, medical treatment, surgery, or public health intervention) under ideal or controlled conditions. Effectiveness is the ability of a [44] medical intervention (e.g., teleconsultation) to have a significant effect on patients under normal clinical conditions. In turn, efficiency must also clearly identify the inputs that are used to obtain the effect of interest (for example, hours of medical care, days when drugs are supplied or medical expenses) [45] Some authors define efficiency as achieving the desired results with the most profitable use of resources [41]. According to Głodziński, efficiency is the achievement of the highest level of satisfaction possible with the given inputs and technologies [46].

The dimensions of medical service effectiveness and efficacy were announced as quality dimensions in the PHC by the WHO Eastern Mediterranean Regional Office (EMRO) [47]. In turn, the efficiency and effectiveness dimensions were proposed by the US Agency for Healthcare Research and Quality (AHRQ) [48]. Effectiveness and efficiency were also used in the tools for quality assessment in Iran’s PHC systems [44].

However, effectiveness is the most popular dimension among the tools for assessing the quality of primary healthcare. For instance, in the Iranian primary healthcare quality assessment framework, (QAF) out of 40 Quality Indicators (QIs), 33.5% were related to the effectiveness dimension. This dimension had the highest share among the quality dimensions [44]. The effectiveness dimension was also in common with the QAF of such countries as Australia, Canada and the United States in terms of the classification of dimensions and QIs [49,50].

Effectiveness plays an essential role in the tools for quality assessments designed for patient opinion surveys. The effectiveness of primary healthcare in regards to obtaining achievable health benefits based on an objective or subjective assessment stating that primary healthcare helped to improve the patient’s health or well-being [51]. Effectiveness in primary healthcare facilities is a set of coordinated actions taken at various levels of reference, improving the patients’ health through prevention and the provision of primary healthcare [52].

Testa and Simonson argue that any area of health can be measured objectively and subjectively [53]. There are therefore two main trends in the literature regarding the measurement of HRQoL. The first one concerns the measurement based on objective indicators, and the second one is based on subjective indicators. While the objective dimension is used to determine the patient’s health status, the patient’s subjective assessment is used to translate this condition into the patient’s actual HRQoL. Hence, two patients with identical health status may have very different HRQoL depending on their subjective experiences, expectations and perceptions of health [5].

Today, most HRQoL tools are based on patient assessments and have a wide range of applications. A key distinguishing feature of HRQoL is the consideration of the patient’s values, judgments and preferences [15,54]. Therefore, literature suggests the construction of social indicators to assess the quality of primary healthcare in a subjective manner [55].

A literature review clearly demonstrated that primary healthcare accessibility and treatment effectiveness are multidimensional constructs. They were taken into consideration in terms of many variables and indicators used to measure them.

In many studies, accessibility has been measured using quantitative indicators that can be objective measures of the availability of primary healthcare. Such objective indicators selected to measure the availability of primary healthcare concern, for instance, the waiting time for an appointment with a specific family doctor, with any family doctor, and for the initiation of consultations [34], the share of people who had or didn’t have contact with the provider at a certain time, or the total number of services provided after contact. Such objective indicators also include the travel time, waiting time in the waiting room, the actual patient consultation time at the medical facility and the weighted sum of the difference between the ideal and the actual number of services, personnel and equipment in the community. In the scale of the entire primary healthcare system, patients’ access to the system can also be measured by the number and availability of primary care physicians (the number of medical personnel, medical facilities per unit of population and per unit of geographic area) [56,57].

As already mentioned, literature suggests the construction of social indicators to assess the quality of medical care in a subjective manner [55]. Subjective accessibility indicators concern the patients’ assessments of various aspects of their experience of being provided with care. Due to the fact that patients play a unique and important role as evaluators of quality of care, it can be concluded that the patients’ opinions should also be taken into account by primary healthcare managers.

Therefore, our tested model provides an accessibility measurement that covers only more subjective indicators related to patients’ opinions regarding access to a primary teleconsultation with a General Practitioner (GP), possibility of contacting a primary healthcare facility via telephone/Internet, possibility of obtaining help in emergency situations, convenient opening hours, punctuality of consultations. Such variable were also used in other studies [34,58,59]. This study did not take into account the accessibility dimensions adapted in terms of residential care, such as the location of the healthcare facility and the person’s ability to access the facility [60], the ease and convenience of reaching a doctor, the availability of services at the place needed [56]. This study also ignores the more detailed accessibility dimensions adopted by Levesque et al. [61], which do not apply to the Polish conditions and the accessibility definition adopted for the purpose of the study. According to these authors, access to healthcare is affected by individual and environmental factors of the healthcare supply-side factors (e.g., approachability; accommodation; affordability) as well as demand-side factors (ability to perceive; ability to seek; ability to reach; ability to pay ability to engage).

The effectiveness is measured most often with indicators based on an objective or subjective assessment of whether primary healthcare has helped to improve the patient’s health or well-being [51]. The most common measures of effectiveness are related to the quality of life, changes in health status, measures of health or well-being, the results reported by the patient, and the patient’s knowledge [51]. Some authors recommend measuring effectiveness based on the skills and competencies of the medical personnel (physician’s ability to make a proper diagnosis and treatment) [62].

The assessment of the treatment effectiveness of can be considered in three dimensions: (1) the health dimension assessed by the mortality and morbidity rates, (2) the satisfaction dimension, defined as the level of meeting the patient’s expectations regarding primary healthcare, (3) the economic dimension regarding the cost of the services provided [63].

The paper is focused on the satisfaction dimension and examines the effectiveness of treatment as measured by patient satisfaction with improving health, solving a health problem and met expectations towards the treatment plan applied. It was assumed in the study that an effective GP helps to solve a health problem and improves the patient’s health condition, and the treatment plan proposed by him or her meets the patient’s expectations and does not require additional appointments with other specialists [62,64].

The study aims to describe patient satisfaction with the access to primary healthcare and treatment effectiveness in the conditions of remote medical care caused by the COVID-19 pandemic. The study is dealing with the subjective assessment of patient satisfaction in two dimensions: access to primary healthcare and treatment effectiveness. 98 patients of primary healthcare facilities participated in the survey. The other part of this paper is structured as follows. Section 2 includes the specification of the applied research methods. The Exploratory Factor Analysis (EFA) and Confirmatory Factor Analysis (CFA) are used to define the remote healthcare quality factors. Section 3 provides the results obtained in the study on patient satisfaction from access to primary healthcare and treatment effectiveness during the COVID-19 pandemic in Poland. It also includes comments on the impact of access to teleconsultations on the treatment effectiveness. Section 4 includes a discussion about the limitations of this study. Finally, the paper also provides conclusions and practical implications.

## 2. Materials and Methods

### 2.1. Methodology

The research methods used in this study included a subject literature analysis and an analysis of the results of own research carried out in Polish healthcare facilities in 2021. The analysis featured the use of descriptive statistics, the Exploratory Factor Analysis (EFA) and the Confirmatory Factor Analysis (CFA) for the development of the relationship between accessibility and effectiveness of telephone consultations. The extracted factors were used to perform a regression analysis to check the impact of accessibility to telehealth consultations on the treatment effectiveness.

According to Hair et al., the number of observations should be ten times higher than the number of variables in the factor analysis model. The minimum acceptable ratio of observations to variables is 5:1. Some researchers accept the ratio of 3:1. The absolute minimum number of observations in the factor model is 50 [65]. In this study, the number of observations is 98. Due to the fact that the 3:1 ratio requirement was met, an EFA analysis was conducted to indicate the variables loading onto two expected latent factors and indicate the initial structure of the factor model. The objective of the analysis was to prepare a questionnaire for a full-scale survey. After the initial EFA analysis, only eight variables were left since the 5:1 sample per item ratio has been satisfied. In addition, a regression analysis was used to evaluate the impact of accessibility on effectiveness.

#### 2.1.1. Population and Data Collection

The study featured a survey conducted on the patients of the CortenMedic primary healthcare facilities. This preliminary study was aimed at preparing and checking the remote healthcare quality research tool—the structured questionnaire. It was important to explore the data structure and prepare a questionnaire for population studies [66]. The response rate meets all survey standards of at least 60% [67]. The questionnaire was assessed in terms of question comprehensibility and difficulty, clarity and ambiguity, length, completion time, and data collection manner [68].

The research was carried out in four primary healthcare facilities in Poland considering the limitations introduced during the pandemic. One of the facilities is located in Radom, a large district city in Poland. The remaining three (Warsaw 1, Warsaw 2, Warsaw 3) are located in Warsaw, the capital city of Poland. The total number of basic care patients registered in these facilities amounts to 46,700.

The data was collected during two sessions: on 25 February 2021–26 February 2021 and 11 March 2021–12 March 2021 through an anonymous survey with closed-ended questions. The randomly selected adult patients, who used telephone consultations during the pandemic, were surveyed using the computer-assisted telephone interviewing (CATI) method; an interviewer presented survey questions to the patients and collected their answers. All patients who agreed to take part in the survey were included in the study. Completed questionnaires were returned to the researchers who conducted the survey. One hundred five patients participated in the study, six patients declined and did not complete the questionnaire. One record was deleted due to more than 20% of missing data. Ninety-eight complete records were included in the study, representing a response rate of 93%. The survey structure presenting the place and date of data collection is shown in Table 1.

#### 2.1.2. Patient Satisfaction Questionnaire

A research tool for patients’ satisfaction survey was developed based on the previous research [69,70,71,72,73]. The survey instrument focused on relationships between variables consisting of two parts: biographical and methodological. The first part contained information about age, gender, marital status, education, place of residence, current occupation and professional activity, including the place and facility in which the survey was conducted. The methodological part consisted of 47 close-end questions pre-assigned to 7 categories. Each question was rated on the five-point Likert scale. The grade of each question scale was described verbally and numerically as follows: 1—I strongly disagree, 2—I disagree, 3—I am undecided, 4—I agree, 5—I strongly agree. The study only presents the accessibility and treatment effectiveness dimensions.

Accessibility variables were established based on the Haggerty et al. paper. The Authors define accessibility as the ease with which patients can obtain the needed care, support and advice from a selected (variable D2) or any (variable D3) primary care physician at a time (variables D4, D5) appropriate for the urgency of the problem (variable D1) [41].

Effectiveness is considered as a subjective assessment of whether primary care physician contributed to improving the patient’s health. Variables E1 to E4 were selected based on the subject literature [35,52,74,75,76,77]. E1 is responsible for the well-being, E2 describes the doctor’s ability to make an appropriate diagnosis, E4 states that this diagnosis could be made without additional consultations with specialists, E3 takes into account the patients’ expectations (E4). The questions regarding both accessibility and treatment effectiveness are shown in Table 2.

#### 2.1.3. Ethics

The survey instrument was constructed based on the literature review. Literature-driven questions were slightly changed for this study. All questions were confirmed by the research team and by two experts from CortenMedic—a healthcare service provider. Detailed information on the purpose of the research and its course was prepared for respondents. During the first stage of the survey, an interviewer read the study rules out to the respondents. The survey was voluntary and completely anonymous. Only adult respondents took part in the survey. Each patient could withdraw from the study at any time or choose not answer all the questions. The completion of a single questionnaire required 20 min on average. The interviewer read all the questions and answers and marked the patients’ responses in the database form one by one. The questionnaire form was anonymous. Patients completed the questionnaire voluntarily. The questionnaire was assessed from an ethical perspective by the Warsaw University of Technology Senate Committee for Professional Ethics.

## 3. Results

### 3.1. Data Analysis

The analysis was conducted using the SPSS v. 27 statistical package (Predictive Solution, Krakow, Poland) and Microsoft Excel 365 (Microsoft, Redmond, WA, USA). Prior to the questionnaires’ statistical analysis, a database of responses was created. Data from the paper-based questionnaires were transferred to a spreadsheet. In the survey, the questions were not deliberately divided into dimensions. The next step was to sort the statements according to the dimensions: accessibility, coordination, comprehensiveness, effectiveness, continuity, communication and experience with the system; the analysis for the purpose of this study covered only two dimensions: accessibility and effectiveness.

**Figure 1:**
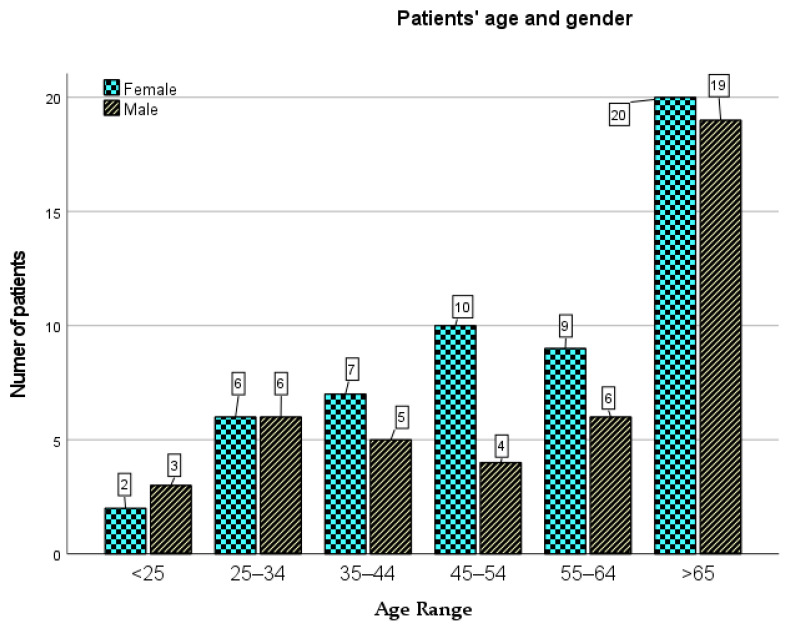
Patients’ age structure by gender.

The respondents were divided into six age groups (Figure 1): aged up to 25 (5 people, i.e., 5.1%), 25 to 34 years of age (12 people, i.e., 12.1%), 35 to 44 years of age (13 people, i.e., 13.1%), 45 to 54 years of age (14 people, i.e., 14.1%), 55 to 64 years of age (15 people, i.e., 15.2%) and aged above 65 (39 people, i.e., 39.4%). One person did not disclose his or her age (i.e., 1%). Eventually, this record was deleted due to many missing data.

The most numerous group included married people (38 people; 38.4%); single persons constituted 28.3% of the respondents (28 persons). There were 20 (20.2%) widowed people and 13 (13.1%) were divorced. In addition, more than half of the respondents were people living in a very large city (with over 250,000 inhabitants)—72 people, i.e., 72.7%, residents of large cities (from 100,000 to 250,000 inhabitants) constituted 19.2% (19 people), medium-sized cities (from 20,000 to 100,000 inhabitants), 3.03% (three persons), rural areas—3.03% (three persons) and small towns (less than 20,000 inhabitants)—2.02% (two persons).

The biggest group included people with higher education—51 people (51.5%), then people with secondary education—26 people (26.3%), the minor groups included people with vocational education—15 people (15.2%) and primary or lower secondary school education—seven people (7.1%). Working people accounted for 44.4% of the population (44 people), retirees and pensioners, also 44.4% (44 people), five people were unemployed (5.1%), also three students were surveyed (3%). Two patients ran their own business (2%), while one person indicated a different economic activity (1%).

Patients who went to a given facility for the first visit (12 people) most often declared that their health condition required occasional visits (seven people); three people felt the need for rare visits, and two people—frequent visits. Control and periodic visits due to treatment continuation or chronic treatment took place once a quarter (10 people), once a month (nine people) or once a year (eight people). At the same time, remote consultations were used several times a month by three persons. The need to consult a GP for prevention and health promotion purposes (including vaccination) was revealed rarely (eight people), sporadically (five people) and often (three people). Twenty three patients asked for a prescription, referral to a specialist doctor or sick leave. The remaining patients met a doctor once a year (seven people), several times a month (six people) and once a month (four people). The surveyed patients most frequently visited the doctor once a quarter (45 people) and once a year (27 people), while the least numerous—once a month (18 people) and several times a month (nine people). During the COVID-19 pandemic, patients do not want to consult doctors unless they have urgent reasons [24].

Ninety four people consulted a doctor via telephone. Most patients were waiting for telephone consultation for more than 48 h (43 people); 28 people consulted a doctor the next day and 23 people—on the same day (including quick visits—11 people and waiting time exceeding 4 h—12 people). Two people used video calls via WhatsApp and Skype, their waiting time for consultation exceeded two days. Two people used Microsoft Teams and Zoom. The waiting time for a telephone consultation exceeding two days resulted in a poor evaluation of the healthcare facility.

#### 3.1.1. Accessibility

The D1–D7 variables presented in Table 2 were used in the assessment of accessibility. The descriptive statistics of these dimension variables are shown in Table 3.

**Figure 2:**
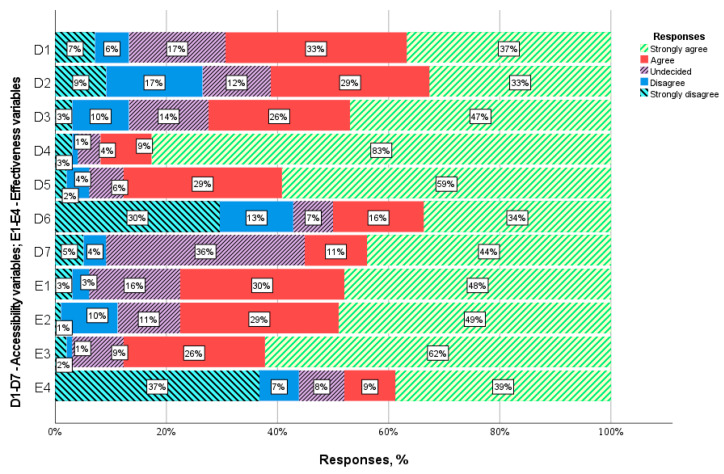
Distribution of accessibility and effectiveness responses.

Patients are most satisfied with the HC facility’s working hours (D4: $\bar{x}$ = 4.68). 91.9% of the respondents claim that the facility’s working hours (from 8:00 to 20:00) are convenient for them. 87.9% of patients are also satisfied with the punctuality of the visits (D5: $\bar{x}$ = 4.38). Unfortunately, 27.3% of respondents have a problem with making an appointment with a GP of their choice (D2: $\bar{x}$ = 3.57) and 13.1%—with booking an appointment with any GP (D3: $\bar{x}$ = 4.03). As many as 42.4% of patients reported that they had a problem with contacting the HC facility via telephone or Internet (D6: $\bar{x}$ = 3.12). 54.5% of the respondents believe that they can easily ask questions after the visit (D7: $\bar{x}$ = 3.85). 35.4% of respondents did not know how to answer question D7 because they have never used this form of contact after the consultation. If they had doubts or wanted to ask the GP additional questions, they made another appointment. 69.7% of the respondents stated that they could obtain medical aid whenever needed, even in an emergency (D1: $\bar{x}$ = 3.86). The distribution of answers is presented in Figure 2.

#### 3.1.2. Effectiveness

The E1–E4 variables presented in Table 2 were used in the assessment of effectiveness. The descriptive statistics of these dimension variables are shown in Table 4.

The overall positive assessment of medical teleconsultations resulted from its high effectiveness. The applied treatment helped (29.3%) and definitely helped (47.47%) improve the respondents’ health. The health problem addressed by 29.3% of patients was partially solved and solved for 48.5% of the respondents. The mean assessment of these variables was similar: (E1: $\bar{x}$ = 4.15) and (E2: $\bar{x}$ = 4.14). The treatment plan proposed by the doctor met the expectations of 26.3% of the patients and complied with the wishes of 61.6% of the respondents. The indications of primary care physicians largely took into account the expectations of patients—this was the best-assessed variable examining the effectiveness of remote consultations (E3: $\bar{x}$ = 4.44). In the patients’ opinion, their health problem did not require (9.1%) and definitely did not require (38.4%) additional medical consultations with other specialists. However, many patients believed otherwise—7.01% disagreed and 37.4% strongly disagreed with this statement. Hence, the health problems reported by half of the patients could be resolved during teleconsultation with a general practitioner (E4: $\bar{x}$ = 3.04). Question E4 was assessed as average by all patients. The distribution of answers is presented in Figure 2.

### 3.2. Factor Analysis

#### 3.2.1. Exploratory Factor Analysis

Exploratory factor analysis was conducted based on 98 observations for two dimensions: accessibility and effectiveness. Variables D4 and D5 did not meet the normality assumption. They were therefore removed from the EFA model. Variable D1 did not load correctly on the expected factors—accessibility. Eventually, eight variables: D2, D3, D6, D7, E1, E2, E3, and E4 were left. The principal component analysis (PCA) and promax rotation with Kaiser normalisation were used to extract two components (Table 5). The Kaiser-Meyer-Olkin measure of sampling adequacy (KMO) equalled 0.73 > 0.6. The KMO value considered as correct is 0.6. Bartlett’s test of sphericity provided a significant result (χ^2^ = 201.125; df = 28, *p* < 0.0001). The probability *p* should be smaller than 0.05, thereby indicating that the values are correct and the sample size is sufficient for the factor analysis.

PCA retained two factors with eigenvalues greater than 1. The total variance explained by the EFA model was equal to 56% (Table 6), which should be greater than 50% [78]. For eight variables, the factor loadings ranged from 0.638 to 0.837 and are greater than the recommended 0.35 cut-off point [79]. A reliability analysis showed that the extracted model was acceptable since the Cronbach’s alpha coefficients for accessibility (0.666) and effectiveness (0.663) were greater than 0.6 [80]. Those values (Table 6) allowed for further factor analysis [65,81].

#### 3.2.2. Confirmatory Factor Analysis

**Figure 3:**
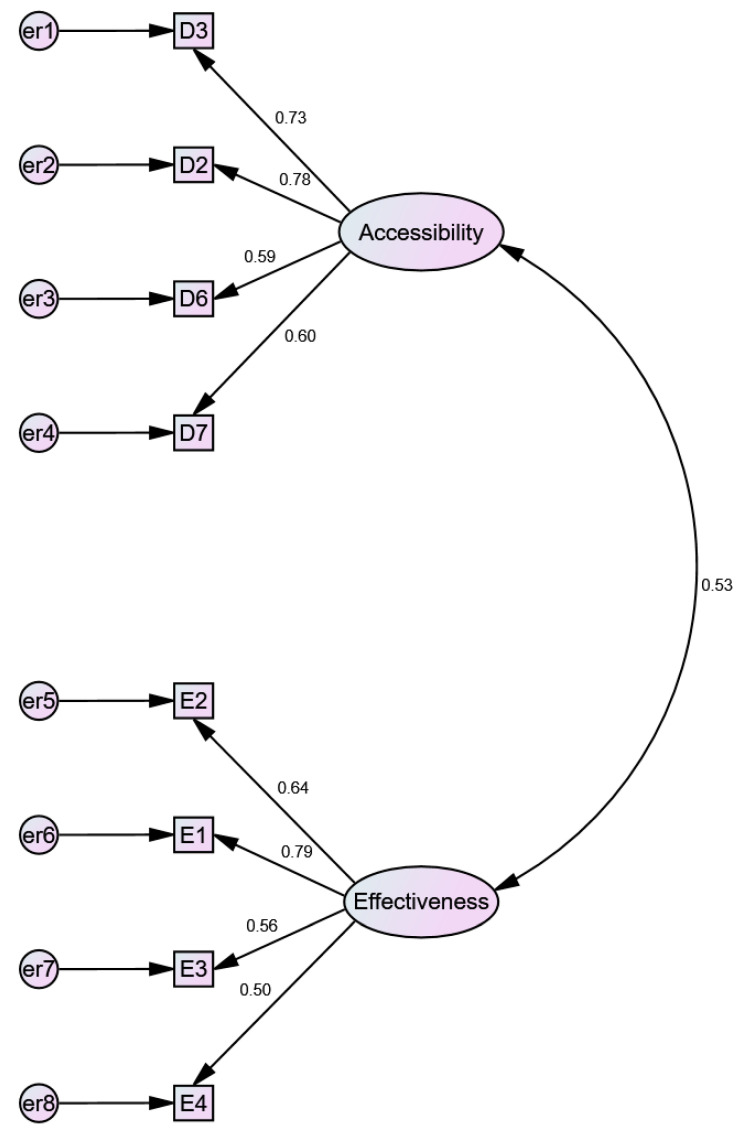
CFA model for the treatment effectiveness and the access to healthcare services during the COVID 19 pandemic (standardised estimates).

**Figure 4:**
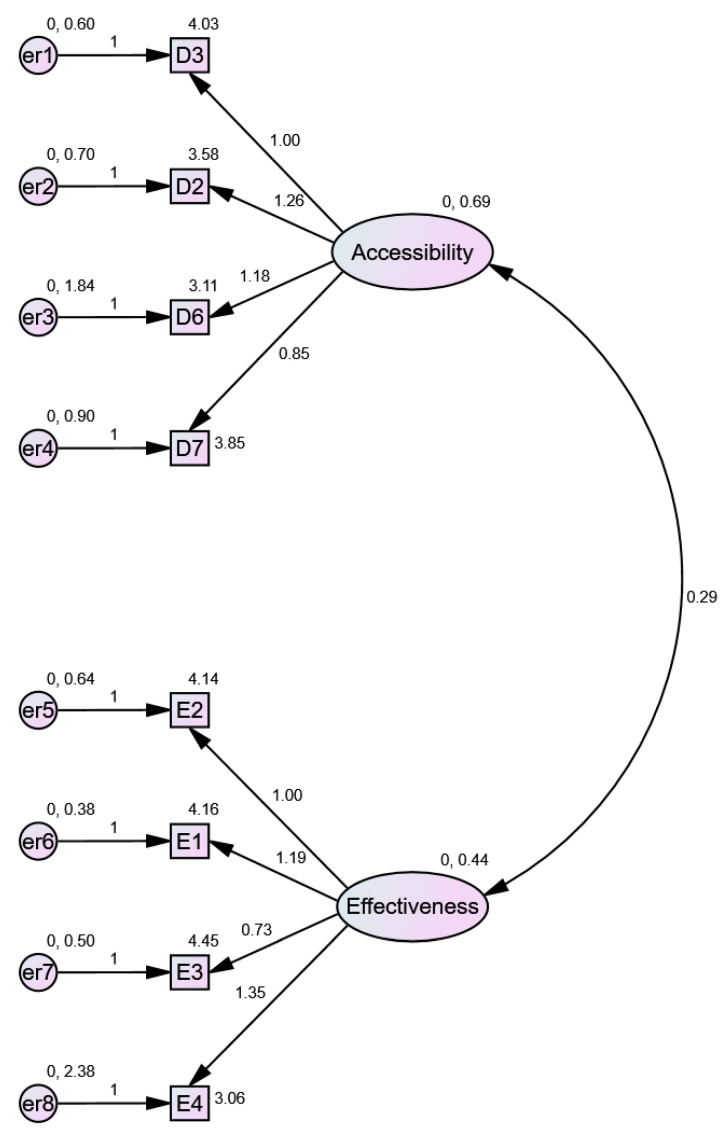
CFA model for the treatment effectiveness and the healthcare services accessibility during the COVID 19 pandemic (non-standardised estimates).

The CFA confirmed the EFA model with eight variables. Standardised and non-standardised solutions are presented respectively in Figure 3 and Figure 4. A convergent validity—the strength of relationships of the model’s factor variables is not supported by the average variance extracted (AVE), which has to be greater than 0.5. The AVE for accessibility equals 0.461 and AVE for effectiveness equals 0,401. This confirmed the low values of the Cronbach’s alpha coefficients calculated during the EFA. Nevertheless, the convergent validity might be confirmed using the composite reliability (CR) index, which should be higher than 0.7. CR values for accessibility and effectiveness equal 0.771 and 0.722, respectively, which means that the convergent validity of the model is confirmed.

The model’s discriminant validity is also confirmed using the Fornell Larker criterion, since the square root of the AVE for each factor is higher than the correlation between factors (Table 7). Also, the hetero trait − mono trait method points to a correlation between factors of 0.539, which should be smaller than 0.85. Taking into account the above results, it is possible to confirm the model’s reliability and validity.

The model fit measures indicate that the model is correct. CMIN = 20.918; DF = 19.000. CMIN/DF = 1.101 (>1; <3), CFI = 0.989 (>0.95); SRMR = 0.054 (<0.08); RMSEA = 0.032 (<0.06); *p*-value = 0.605 (>0.05).

#### 3.2.3. Regression Analysis

Aside from the CFA model, a regression analysis was also conducted. Two variables for effectiveness and accessibility were calculated based on the CFA model. Two hypotheses were formulated: null hypothesis H_0_ stating that there is no statistical relationship between effectiveness and accessibility variables and H_1_ hypothesis assuming that accessibility affects effectiveness.

**Hypothesis** **H_0_.** *Accessibility does not affect effectiveness (null hypothesis).*

**Hypothesis** **H_1_.** *Accessibility affects effectiveness (alternative hypothesis).*

The correlation analysis pointed to a relationship between the two variables. The Pearson Correlation equals 0.39 and is significant (Table 7), thereby allowing for regression analysis.

The ANOVA analysis showed that F coefficient equals is significant, F (1; 96) = 17.23; *p* < 0.001 (Table 8). The regression model points to the explanation of 14.3% of the variance, i.e., adjusted R-square equals 0.143 (Table 9). In the regression equation (Equation (1)), the constant is insignificant since the relationship between the variables can be described as follows:

Accessibility (±0.93) = 0.39 × Effectiveness (±0.094)(1)

Based on the above regression analysis H_0_ hypothesis was rejected in favor of the alternative hypothesis H_1_.

## 4. Discussion

The quality of care in primary healthcare is a very important element of QOL and represents a combination of many dimensions, including access to healthcare and treatment effectiveness. Papers examining the full spectrum of dimensions of the quality of primary healthcare constitute important diagnostic tools in a health policy. As already mentioned, a well-organised and effective primary healthcare is able to respond to 80% of health needs, that is why it is so significant to medical care.

The first aim of the study was to identify variables for measuring access to primary healthcare and treatment effectiveness in primary healthcare units. The conducted literature analysis was aimed at suggesting appropriate initial sets of indicators for the assessment of access to primary healthcare and treatment effectiveness in remote conditions. The conducted statistical analyses were aimed at reducing and improving the critical empirical indicators used to measure the analysed constructs. Using various data reduction methodologies, the paper’s objective was to identify a basic set of variables that could effectively measure the dimensions of remote primary healthcare accessibility and treatment effectiveness. The objective was achieved in the study. Referring to previous studies, seven variables were originally selected to measure the access do teleconsultations [34,41,58,59] and four variables to measure the treatment effectiveness [35,52,74,75,76,77]. As a result of a factor analysis, the number of variables to measure the access was reduced to four. The Exploratory Factor Analysis showed that the final model adopted for further research was correct.

The second aim was to study patients’ satisfaction with these two dimensions of quality of primary healthcare and to analyse the impact of access to primary healthcare on the treatment effectiveness during the COVID-19 pandemic in Poland.

Most of the patients in this study positively assessed the access to primary healthcare and treatment effectiveness in the conditions of teleconsultation in primary healthcare facilities during the COVID-19 pandemic in Poland. This was the case even though telemedicine was never used in the Polish primary healthcare institutions before. 55.5% of the respondents believe that the medical care they received during teleconsultation was as good as meeting their GP face to face [82] The previous studies confirmed that, for some patients, telehealth can be as good as or even better than personal care, especially for those faced with problems concerning physical appointments, e.g., people living in rural areas [83].

According to the results, patients are rather satisfied with the access to remote primary healthcare. The vast majority of patients agree or strongly agree with all positive aspects of the care accessibility dimension. They are the least satisfied with the possibility of contacting the clinic via telephone/Internet (D6: $\bar{x}$ = 3.12) and the possibility of making an appointment with the GP of their choice (D2: $\bar{x}$ = 3.57). On the other hand, the clinic’s working hours are rated the highest (D4: $\bar{x}$ = 4.68). Majority of respondents stated that they could obtain medical help whenever needed, even in an emergency (D1: $\bar{x}$ = 3.86). Quick access to GP appointments was assessed more positively in previous studies in which quick emergency care accessibility was rated the highest [84,85].

Systematic studies have shown that telemedicine has already been successfully used in other countries to provide routine and specialist medical services and has led to greater access to medical care. Moreover, telemedicine has shown similar, and in selected circumstances better, health effects compared to the conventional models of care [83,86], while demonstrating the ability to reduce unnecessary hospitalisations and costs [87].

In general, an analysis of other studies shows that telemedicine is actually pursuing its primary goal of improving access to care and it does so through innovative and constantly evolving tools. For instance, in Great Britain and Denmark, in order to ensure access to primary healthcare, teleconsultations take place in most primary care facilities as a standard procedure [88,89,90]. It is treated as “a strategic alternative to decentralisation and improving access to medical care, allowing to reduce costs and travel time for patients” [88].

Looking at the benefits, teleconsultations can reduce the patients’ indirect costs in terms of time and money, and increase access to primary healthcare, especially if telehealth can be used to support routine or stable patient health problems [91]. The other benefits include less need for face-to-face consultations, the ability to manage physician workloads and allowing systems to be reorganised [88]. In addition, teleconsultation enable overcoming the distance barriers in a flexible and convenient way for patients, with the ability to contribute to the continuity of care, patient autonomy and resource savings. Other qualitative studies examining satisfaction with teleconsultation show that the main benefits commonly reported by patients are convenience, reduced travel time and precisely greater access to specialist care, as well as better appointment flexibility, enabling minimal disruption of everyday life [92,93].

Teleconsultations cannot replace personal medical care in all cases. Several studies have shown that patients were satisfied with the remote consultations, but would also like to be able to have face-to-face appointments [83]. The teleconsultations should not be used in rare or unstable conditions, or when a physical examination is needed. Some patients are more appreciative of direct contact with a physician (with direct examination if necessary) compared to the convenience of telehealth, which was also confirmed by previous studies [94,95,96]. Such direct contact is also necessary in the case of seriously ill patients. Unfortunately, this pilot study did not allow for the assessment of the quality of teleconsultation from the point of view of chronically ill people. Most of the respondents (40.4%) contacted a doctor for non-urgent reasons (administrative matters: prescription, referral to a specialist, sick leave) or for control reasons (30.3%). Only 12.1% of the respondents held a teleconsultation due to chronic treatment. The other studies show that it is important for the patients to have the choice and flexibility to use health services in the most appropriate way [97]. However, it should be remembered to enable personal appointments for people with more complex health needs [98].

The overall positive assessment of the quality of remote primary healthcare was also due to its high treatment effectiveness. Most patients participating in this study rated the treatment effectiveness quite high. The treatment applied helped or definitely helped most patients, as most of them have had their health problems solved (E1: $\bar{x}$ = 4.15). The patients’ expectations were taken into account by the GPs in the majority of cases (E3: $\bar{x}$ = 4.44). Therefore, teleconsultations seem to be a safe and effective way of assessing and dealing with various clinical situations.

Also, other studies confirm that telemedicine maximises primary healthcare and offers the possibility of improving the treatment effectiveness [99]. The support for new communication technologies in the healthcare service provision is an important determinant of quality sought by all participants. Technological advances that are transforming traditional treatments and modern methods of care and diagnostics lead to positive changes in the form of better treatment outcomes for patients living in developing, rural areas or areas with limited healthcare options [100,101].

The correlation analysis and ANNOVA analysis conducted in this study pointed to a relationship between access to healthcare and treatment effectiveness in the primary healthcare. The regression model indicated that 14.3% of the variance is explained. The literature also consistently indicates that in the case of some diseases, telemedicine leads to the improvement of health outcomes. In areas such as type 2 diabetes, research shows that telemedicine intervention is comparable to the standards of traditional medical care and does not cause unnecessary risk or harm to patients [102]. Also, neurological and cardiological signs and simple ophthalmic symptoms such as strabismus can be safely diagnosed and treated through teleconsultation [83,88]. However, this study is the first one to show the impact of medical care accessibility on treatment effectiveness in primary healthcare in a crisis situation, such as the COVID pandemic.

There are some limitations to this study that need considering. Firstly, the indicators for measuring medical care accessibility and treatment effectiveness, despite their validation, have not been used in other populations, and therefore their external validity has not yet been confirmed. The same limitation can be attributed to the study population, which despite a large size is derived solely from four entities located in one region and therefore must be generalised conservatively. Although the sample size of the patients studied was varied, the extent to which they are representative of patients in other clinics is unknown. It would be interesting to repeat the study in other healthcare entities or organisations to see if these variables do indeed still determine the ultimate quality of care based on access to healthcare and treatment effectiveness. Otherwise, the studies conducted among primary care patients in Europe suggest that interpersonal aspects (e.g., communication with a physician, trust and respect [34] are more important dimensions of healthcare quality than accessibility and effectiveness, thereby making it necessary to include them in future studies. It is fair to say that research into these relationships requires further attention. However, the deliberations are limited solely to primary healthcare. Patients with severe diagnoses, e.g., cancer or unstable chronic diseases, would probably assess healthcare services differently than patients requiring stable follow-up appointments, thereby requiring more attention to be paid to groups of patients with unstable health conditions.

## 5. Conclusions

The COVID-19 pandemic has disrupted the provision of healthcare services, resulting in a considerable deterioration in patients’ overall health, especially those with chronic diseases. Broad access to telemedicine could significantly reduce disruptions in the provision of healthcare services during the pandemic and prevent, at least to some extent, a deterioration in the patients’ quality of life and careers. While remote healthcare solutions cannot completely replace face-to-face medical assessment, they can ensure the continuity of healthcare services and help protect patients, their families and healthcare professionals from disease transmission. The accessibility and effectiveness of medical care are considered key features of the care processes required to ensure high-quality outcomes. Sufficient documentation of the relationship between the accessibility and effectiveness of patient care is essential to support efforts to improve the outcomes of all types of disease treatment, especially chronic disease, and finally to improve the patients’ quality of life. The purpose of this study was to examine patient satisfaction with the access to primary healthcare and the effectiveness of treatment in a condition of remote medical care caused by the COVID-19 pandemic. The model proposed in this study identified a positive and weak, but statistically significant, relationship between these factors. Although a better access to primary care has a positive effect on treatment effectiveness, there are undoubtedly other factors that affect this effectiveness to a greater extent and it would be worth investigating them in further studies.

Taking into account patients’ views on the quality of medical services can help to improve overall healthcare delivery in the primary healthcare that is responsible for most health needs. Improving the quality of this care is of great clinical importance. It can positively impact the early detection of chronic diseases, rapid, effective and patient-centered delivery of medical care, adherence to treatment protocols and thus clinical outcomes. As a result, it can also lead to a better quality of life for patients. In addition, tools in the field of telemedicine, implemented at the primary healthcare level, can support clinical decision-making and thus improve the effectiveness of care by providing healthcare professionals with information and knowledge about a specific patient at the right time during interaction with the patient. According to the WHO, this can promote effective decision-making and enable different healthcare providers to understand and deal with the broad and complex health problems encountered in primary care.

To our knowledge, this is the first study in which access to this healthcare was examined on a sample of Polish primary care patients in the conditions of remote work caused by the COVID-19 pandemic. An analysis of the data from this study showed that patients positively assessed the accessibility of remote services and the treatment effectiveness in teleconsultation conditions. Future research should therefore be focused on patients with chronic diseases requiring coordinated healthcare and should also be extended to outpatient healthcare facilities. Health variables should also be considered as moderating variables in future studies.

## Figures and Tables

**Table 1:** Structure of the survey responses.

		Date and Type of the Survey		Total
		February 2021 CATI	March 2021 Paper-Based Survey	
		Number of Responses		
Facility	Warsaw 1	0	21	21
	Warsaw 2	19	0	19
	Warsaw 3	22	19	41
	Radom	24	0	24
Total		65	40	105

**Table 2:** Questions concerning the accessibility and treatment effectiveness dimensions initially included in the questionnaire.

Variable Name		Question
Accessibility	D1	I can get medical help when I need it, even in case of emergency
	D2	I can easily make a telehealth consultation with a General Practitioner (GP) of my choice
	D3	I can easily make an appointment with a GP at the healthcare facility
	D4	The healthcare facility’s working hours are convenient
	D5	Telehealth consultations take place at an agreed time
	D6	I can easily contact the healthcare facility via phone / Internet
	D7	I can easily ask questions after the telehealth consultation
Effectiveness	E1	The treatment helped me improve my health
	E2	The health problem with which I turned to the GP was solved
	E3	The treatment plan proposed by the GP meets my expectations
	E4	The health problem with which I turned to the doctor did not require additional medical consultations with other specialists

**Table 3:** Descriptive statistics of Accessibility variables.

Variable	Mean				Skewness		Kurtosis	
	Statistic	Std. Error	Std. Deviation	Variance	Statistic	Std. Error	Statistic	Std. Error
D1	3.8571	0.12049	1.19276	1.423	−0.983	0.244	0.202	0.483
D2	3.5816	0.13601	1.34642	1.813	−0.565	0.244	−0.966	0.483
D3	4.0306	0.11554	1.14382	1.308	−0.989	0.244	−0.023	0.483
D4	4.6735	0.08676	0.85886	0.738	−3.098	0.244	9.634	0.483
D5	4.3878	0.09359	0.92650	0.858	−1.807	0.244	3.223	0.483
D6	3.1122	0.17032	1.68610	2.843	−0.127	0.244	−1.703	0.483
D7	3.8469	0.11992	1.18715	1.409	−0.603	0.244	−0.505	0.483

**Table 4:** Descriptive statistics of effectiveness variables.

Variable	Mean				Skewness		Kurtosis	
	Statistic	Std. Error	Std. Deviation	Variance	Statistic	Std. Error	Statistic	Std. Error
E1	4.1633	0.10225	1.01223	1.025	−1.249	0.244	1.276	0.483
E2	4.1429	0.10560	1.04536	1.093	−1.066	0.244	0.148	0.483
E3	4.4490	0.08718	0.86301	0.745	−1.902	0.244	4.127	0.483
E4	3.0612	0.18106	1.79240	3.213	−0.072	0.244	−1.817	0.483

**Table 5:** Pattern matrix for the EFA model.

Variable	Component	
	Accessibility	Effectiveness
D3	0.804	
D6	0.795	
D2	0.777	
D7	0.686	
E2		0.837
E1		0.795
E3		0.645
E4		0.638

Extraction Method: Principal Component Analysis. Rotation Method: Promax with Kaiser Normalization, a. Rotation converged in 3 iterations.

**Table 6:** Eigenvalues and total variance explained by the EFA model.

Component	Initial Eigenvalues			Extraction Sums of Squared Loadings			Rotation Sums of Squared Loadings
	Total	% of Variance	Cumulative %	Total	% of Variance	Cumulative %	Total
1	3.185	39.809	39.809	3.185	39.809	39.809	2.711
2	1.374	17.178	56.987	1.374	17.178	56.987	2.551
3	0.841	10.512	67.499				
4	0.725	9.069	76.568				
5	0.564	7.055	83.623				
6	0.527	6.594	90.216				
7	0.474	5.921	96.137				
8	0.309	3.863	100.000				

Extraction Method: Principal Component Analysis.

**Table 7:** Correlations between accessibility and effectiveness.

Correlations				
		D_Accessibility	E_Effectiveness	AVE
D_Accessibility	Pearson Correlation	1	0.390 **	0.461
	Sig. (2-tailed)		0.000	
	N	98	98	
E_Effectiveness	Pearson Correlation	0.390 **	1	0.401
	Sig. (2-tailed)	0.000		
	N	98	98	

**. Correlation is significant at the 0.01 level (2-tailed).

**Table 8:** Correlation between accessibility and effectiveness.

ANOVA ^a^						
Model		Sum of Squares	df	Mean Square	F	Sig.
1	Regression	14.762	1	14.762	17.233	0.000 ^b^
	Residual	82.238	96	0.857		
	Total	97.000	97			

^a^ Dependent Variable: E_Effectiveness. ^b^ Predictors: (Constant), D_Accessibility.

**Table 9:** Regression model summary.

Model Summary ^b^				
Model	R	R Square	Adjusted R Square	Std. Error of the Estimate
1	0.390 ^a^	0.152	0.143	0.92554997

^a^ Predictors: (Constant), D. ^b^ Dependent Variable: E.

## Data Availability

Data is contained within the article.
